# Changes In Mental Health, Social Engagement, and Physical Activities During the COVID-19 Pandemic

**DOI:** 10.1093/geroni/igab046.541

**Published:** 2021-12-17

**Authors:** Wenjun Li, Su-I Hou

## Abstract

The current COVID-19 pandemic has profoundly changed our behaviors and health, especially vulnerable community-dwelling older adults. This symposium includes three presentations that evaluated the pandemic’s impacts on mental health, social engagement and physical activity in healthy community-living older adults and those with dementia. Dr. Wenjun Li and his team examined the pandemic impact on mental health and social engagement among relatively healthy older adults residing in suburban and rural neighborhoods in Central Massachusetts, USA. The study reported significant variations in pandemic impacts by sex, age, race, income, living arrangement, and neighborhood housing density, suggesting the pandemic has had disproportionally negative impacts on socially and economically disadvantaged vulnerable older adults. Dr. W. Quin Yow and her team evaluated the impacts of government mandated social distancing and lockdowns on older adults with dementia and their caregivers in Singapore. The study found significant increases in irritability, aggression and hallucinations among older adults with dementia, and possible deterioration of health conditions and heightened moderate level of stress. The results suggest that social distancing may have resulted in negative outcomes in this vulnerable population with dementia and their caregivers. Dr. Ladda Thiamwong reported her team’s efforts on forming an international aging research collaborative to mitigate heath consequences of COVID-19 crisis from the international perspective. The team consists of ten scholars from five countries, including Hong Kong, Nepal, Singapore, Thailand, and the United States. They collect data using combinations of online and face-to-face surveys. Their important findings will be discussed in detail in this symposium.

